# Gender preference and fertility behavior among married women: A community based study from far western Nepal

**DOI:** 10.1371/journal.pgph.0001080

**Published:** 2024-06-06

**Authors:** Pratima Dawadi, Aarati Sharma Bhatta, Laxmi Rajbanshi, Rajendra Gautam

**Affiliations:** 1 Research Section, Nepal Health Research Council, Kathmandu, Nepal; 2 School of Nursing, Chitwan Medical College, Bharatpur, Chitwan, Nepal; 3 Department of Microbiology, Peoples Dental College and Hospital, Nayabazar, Kathmandu, Nepal; PLOS: Public Library of Science, UNITED STATES

## Abstract

Gender preference often results in low use of contraceptives and parity progression, which can increase the risks of morbidity and mortality for women. This study aimed to identify gender preference and fertility behavior, including contraceptive use and the desire for additional children, among married women. A cross-sectional descriptive study was conducted using systematic random sampling to select280 household, with one respondent interviewed from each household using a semi-structured interview schedule. Descriptive and inferential statistical analysis were performed on the collected data. Of 280 respondents, 44.6% were aged 26–35 years (mean30.23±7.39 years). Most (74.3%) were literate, and 70% were paid worker. Son preference was reported by 53%, with support in old age (87.2%) being the main reason. Gender preference was 60.5% less likely among Bramhin/Chhetri ethnic groups (p = 0.033) and 71.3% less likely if husbands were literate (p = 0.002). Contraception use was 90.7%, but 31.8% desired additional children. Permanent contraceptive method use was 9.387 times more likely above age 30 years (p = <0.001), independent of respondents’ and husbands’ education, sex composition of children, and having a preferred child. Desire for more children was 6.813 times more likely below age 30 years (p = <0.001) and 5.875 times more likely with 1–2 living children (p = 0.001), independent of respondents’ and husbands education.The study concludes that son preference persisted among the illiterate. Contraceptive use was lower among respondents below 30 years. Enhancing educational status may reduce gender bias. Targeting family planning to women below 30 years could improve contraceptive utilization in this age group.

## Background

Gender refers to the socially constructed characteristics, norms, roles and relationships that exist between women and men. These gender expectations vary among cultures and can change over time [1].

The term “gender preference” describes the desire of biological parents for either a male or female child and in extreme cases the use a range of unethical methods (e.g., infanticide and sex-selective abortions) to achieve that result. In patrilineal societies, the necessity for a male birth is straightforward. Less-developed societies tend to favor males for immediate utilitarian reasons and, eventually, as a primitive social security system [2].

Gender preferences for children have been widely observed around the globe. A balanced preferences is found in Indonesia, France and Poland whileLatin American and Caribbean countries exhibit a marked daughter preference [3]. Similarly,inthe Dominican Republic (34.5%) and Haiti (23.2%), women have a higher preference for daughters.On the other hand, North African and South Asian countries are marked by strong preference for sons [3].

Daughter are married and sent to their husband’s house, and because of the dowry system, which requires the bride’s family to give the groom durable goods as a condition of marriage, daughters are viewed as an economic responsibility [4].Son preference and neglect of girlsoccur even among the educated and affluent classes in India and are not correlated with economic development, affluence, or literacy levels. The low status of women and patriarchal values are intensifying this trend in India. Son preference has serious negative effects on women’s health, fertility choices, and future wellbeing of girls [5].Sons are preferred because they are seen as conferring social value upon their family, carrying the family name, inheriting, and shouldering economic responsibility [6].Son preference is a common phenomenon in Nepal. Having a son is essential for several reasons. Sons remains in the family, so they can support their parents in old age. Various religious and cultural functions can only be performed by the sons, such as *Sraddha*, the ritual performed to pay homage to one’s ’ancestors’ especially to one’s dead parents [7].

Male roles are differently valued than female roles both roles serve a function, but there is a dominance in value attributed to the role of men and sons in society. Women are considered an economic burden, while sons are considered assets. Inheritance and land rights favor males and ageing parents depend on their sons for elderly care. The higher participation of males in formal income-generating activities place them in positions of power within the family. Moreover, some religious rituals can only be performed by sons, such as funeral rituals upon a parent’s death. Women, on the other hand, leave their parents’ home upon marriage and take dowries, making them a perceived unproductive investment from the parents’ perspectives [8].

Strong son preference, compounded by availability of sex determination technology, enables couples to resort to sex selective abortion, which contributes to increased Sex Ratio at Birth (SRB) and an imbalanced population sex ratio [9]. Son preference has implications for women’s health, fertility and well-being. Women have to face multiple pregnancies, abortion and infanticides to have a son [10]. Women face violence and humiliation from their husband and in-laws, negativelyimpactingtheir physical and mental health and resulting in the neglect of infant girls [5].Sex selection is an overt manifestation of son preference that involves taking action to ensure the birth of a boy, prevent the birth of a girl, and in some extreme cases, allow or cause the death of a girl child.The missing population of girls due to strong son preference in countries like India, China and Nepal is known as “Asia’s missing women” [11–13]. Sex selective actions can be taken before pregnancy, during pregnancy, and after birth, resulting distorted sex ratio at birth [14].

Nepal’s Sex ratio at birth (SRB)increased significantly from, 103.5 in 1991 to 106 in 2011, while the fertility rate declined from 3.1 in 2006 to 2.6 in 2011. According to the data, the number of male children below 10 years of age in Nepal exceeds the number of female children by 2.2 percentage [15], which might be due to strong son preference. The total fertility rate for women of reproductive age in Nepal fell from around 6 children per women to 3.1 in 2006, 2.6 in 2011, 2.3 in 2016 and 2.1 in 2022 [16].As fertility decreases in Nepal, the impact and effect of gender preference for children become more visible in couple’s reproductive behavior and choices. This combination of positive and negative conditions for fertility decline creates conflicting needs: people desire fewer children but remain concerned about the importance of having a son to carry on the family [17].

A study conducted in five districts of Nepal revealed women face tremendous pressure from husbands (42%) and mothers-in-law (41%) to bear sons. Women who had daughters as their first children faced different forms of physical and psychological violence, such as pressure to bear male children (that included hitting/beating (18%), receiving improper meals (38%), husband’s threatening polygamy (40%) and continuous scolding (86%)). The study found that 8.86% women had used measures to influence the sex of their child to be male, which included observing religious rites, visiting shamans or consulting a doctor to find out if any medical measures could help them to bear male children in their next pregnancy [18].A study conducted in Eastern Nepal revealed that there was a significant relationship between contraceptive use and respondents’ ages and educational levels. The desire of other children was considerably enhanced when there were only female children in the home (AOR = 10.153, 95% CI = 2.357–43.732) [17].

Gender preference for a male child is a well-known phenomenon worldwide. It has multidimensional consequences in the form of imbalanced sex-ratio, sex-selective abortion, female feticide and even female infanticide, violence against women in various forms, violation of rights of women, low contraceptive use and high fertility rates. All these factors place women to higher risk of mortality and morbidity [19]. Hence, this study was carried out to find the gender preference and fertility behavior of married women in a community of far western Nepal.

## Methods

### Ethical consideration

Research approval was taken from the Thesis Committee of Nursing Program, Chitwan Medical College Pvt. Ltd.Ethical clearance was taken from the CMC-IRC, Bharatpur, Chitwan. Formal permission was taken from District Public Health Office (DPHO) Kanchanpur and VDC office Baise Bichawa. Written informed consent was obtained from each respondent by clarifying the purpose of the study prior to the data collection. Respondents’ dignity was maintained by giving option to discontinue their participation in the research study on their will. Confidentiality was maintained by not disclosing the information and assigning code numbers instead of name in the response questionnaire and during data entry.

### Research design

A cross sectionalstudy design was used to determine gender preference and fertility behavior among married women of reproductive age in BaisiBichawa Village Development Committee (VDC), Kanchanpur district, far western Nepal.

### Research setting and population

This study was conducted in Baise BichawaVillage Development Committee VDC [20], now known as Laljhadi Rural Municipality, in Kanchanpur district, Nepal. It is located 60 km from the district headquarter, Mahendranagar, and is one of the remote areas where agriculture is the main occupation. In this VDC, almost 55% female are married at the age between 15–19 years and have an average of 3 children. 20% of women in the age group 40–44 give birth, and the contraceptive prevalence rate is 45%. The community comprises a mixed Ethnicity including Tharu, Chhetri, Dalit, Janajati, Brahmin and Thakuri people, providing an opportunity to study this diverse group in a single study. The study population consisted of married women of reproductive age group 20–49 yrs. Women with at least one living child, currently living with their husbands, and available at the time of data collection were taken for this study.

### Sampling procedure

A systematic random sampling technique was used to select the sample. The first sample was selected randomly by spinning abottle at the junction of the road of ward number, 1 and the sample was chosen where the opening of the bottle pointed. Other samples were taken subsequently at the interval of 9 households. Only one sample was taken from each household. If more than one sample was available in the household, the lottery method was adopted among them to select a single sample unit. If the respondent was not available in the household nearby household (to the left side) was taken to obtain the sample.

### Sample size estimation

The required sample size for this study was calculated to be 280 using the formula for cross sectional survey and applying finite population correction. The non-response rate of 10% was assumed.

We have,

n=Z2pq/d2

Here,

Probability gender preference reduces contraceptive use (p) = 0.24 [21].


q=1−p=0.76

Z = 1.96 for 5% level of significance

Allowable error (L) = 5%

Now, n_0_ = 1.96 × 1.96 × 0.24 × 0.76 ÷ 0.05 × 0.05 = 280

The estimated target population of married woman of reproductive age (15-49years) was 2551 (HMIS 2072/73) andthetotal household in the VDC was 2,593 as per VDC profile of BaisiBichawa (2072).The total households in the VDCwas the sampling frame for the study.

Then,

Required sample size (n) =

$$n=\frac{n_{0}}{1+\frac{n_{0}}{N}}=255(\text{Cochran},1909)$$

Non response rate = 10% of 255 = 25

Total sample size = 255+25

 = 280

Now,

**Table:** Estimating household for each ward using the formula = Total sample size÷Total household of the VDC× Total household of the ward.

WARD	TOTAL HOUSEHOLD	Proportion (%)	ESTIMATED SAMPLE SIZE
1	270	10.41	30
2	123	4.74	13
3	91	3.50	10
4	195	7.52	21
5	539	20.78	58
6	394	15.19	43
7	207	7.98	22
8	226	8.71	24
9	548	21.13	59
Total	2593	100	280

Married women of 15–49 years who had at least one child and who were currently living with their husband were taken as one sample unit and each unit was randomly selected using systematic random sampling technique, at an interval of 9 (Kth item) = N÷n = 2593÷280 = 9^th^ item until the desired sample size was met.

### Research instrument

A semi-structured interview questionnaire was developed following a thorough literature review. The questionnaire had 2 parts:

Part I: Questions related to Socio-demographic characteristics.
Part II: Questions related to gender preference and fertility behavior.

The research instrument was translated into Nepali language with the help of a language expert after content validation via expert consultation.

The research instrument was pre-tested in 28 samples in a similar setting of Rajhar VDC of Nawalparasi district, and modificationsweremadeaccordingly after consultation with the advisor and subject experts. Question number 18, related to child preference, and question number 20, related to reasons for daughter preference, were modified. Furthermore, the questions were rearranged for sequential flow of the interview session.

### Data collection procedure

After obtaining approval from all concerned authorities, including the respondents, data were collected using face to face interviews.The objectives of the study were explained to each respondent, and the interviews were conducted in a private corner of their house or their preferred area.Data collection was done from March, 2017 to January 2018.

### Data analysis procedure

The collected data were checked, edited and organized manually each day. The data derived from semi-structured interview was coded and entered in Epi data3.1. The entered data were exported into IBM SPSS 20 version. The data were analyzed according to the nature of the variables by using descriptive statistics (percentage, mean, standard deviation) and inferential statistics (binary logistic regression, multivariate logistic regression) to test the association between the variables.

## Result

### Socio-demographic characteristics, gender performance and fertility behavior of the respondents

Out of 280 respondents 44.6% of the respondents were in the age group of 26–35 years and 2.1% were in the age group 46 years and above. Mean age of respondents was 30.23±7.39. As per ethnicity 57.9% were Janajati and 0.4% was Muslim. Concerning to monthly income only 8.9% respondents had monthly income 20000 and above. Likewise, out of 280 respondents 41.1% had two children and 28.2% had one child. Regarding sex composition of children among respondents who had 2 and more than two children, 67.2% had both son and daughter, on the other hand had only 12.9% had only daughter. Concerning to educational status around one third (25.7%) of respondents were illiterate and only 1.4% respondents had educational level bachelor and above. Similarly, around one ten (10.7%) respondent’s husbands were illiterate and more than three ten (35.7%) husbands had basic level of education. On the other hand, majority of respondents (70%) were paid workers and 100% husbands were paid workers (Table 1).

**Table 1:** Socio-demographic characteristics of the respondents.

Characteristics	Number	Percentage
**Age (years) (n = 280)**		
16–25	88	31.5
26–35	125	44.6
36–45	61	21.8
46+	6	2.1
Mean±SD = 30.23±7.39		
**Ethnicity (n = 280)**		
Janajati	162	57.9
Brahmin/Chhetri	66	23.6
Others	52	18.5
**Educational status of respondent (n = 280)**		
Literate	208	74.3
Illiterate	72	25.7
**Educational status of respondent’s husband (n = 280)**		
Literate	250	89.3
Illiterate	30	10.7
**Monthly income of family (NRs) (n = 280)**		
< 10000	99	35.4
10000–20000	156	55.7
≥20000	25	8.9
**Number of living children (n = 280)**		
≤2	194	69.2
>2	86	30.7
**Sex composition of children ≥ 2 (n = 201)**		
Both	135	67.2
Only sons	40	19.9
Only daughters	26	12.9
**Occupation of the respondent (n = 280)**		
Paid work	196	70.0
Unpaid work	84	30.0
**Occupation of the respondent’s husband (n = 280)**		
Paid work	280	100

*** Multiple responses

### Gender preference of respondents’

Out of 280 respondents, more than half (52.9%) preferred son and 15.4% preferred daughter. Likewise, among those who reported preference 75.9% had child of their preferred gender and 24.1% did not had child of their preferred gender. Similarly, 47.9% respondents wanted their first child to be a son and 15.7% wanted daughter as their first child. Likewise, 87.2% respondents preferred son for support in old age and 2% for obtaining dowry and 76.7% respondents preferred daughter for enjoying festivals and 2.3% for help in childcare. Regarding current use of contraception majority (74.6%) respondents were using temporary method of family planning and most common method was Depo-Provera (35.6%), which was alike national data. Regarding desire for additional children, 68.2% women had no desire for additional children and 31.8% had desire for additional children. Among those who desire for additional children more than half (61.8%) respondents had desire for son and 28.1% desired for daughter (Table 2).

**Table 2:** Gender preference of respondents’.

Gender Preference	Number	Percentage
**Preferred gender for child (n = 280)**		
Son	148	52.9
Daughter	43	15.4
No preference	89	31.8
**Have preferred child (n = 191)**		
Yes	145	75.9
No	46	24.1
**Gender preference for first child(n = 280)**		
Son	134	47.9
Daughter	44	15.7
No preference	102	36.4
Reasons for son preference*** (n = 148)		
Support in old age	129	87.2
Social Status	70	47.3
Practical help in day to day life	53	35.8
To perform funeral rites	51	34.5
Land/property inheritance	48	32.4
For obtaining dowry	3	2.0
Reasons for daughter preference*** (n = 43)		
To enjoy festivals	33	76.7
Help in household chores	24	55.8
For companionship	24	55.8
Help in childcare	1	2.3
**Current use of contraceptive (n = 280)**		
No	26	9.3
Temporary	209	74.6
Permanent	45	16.0
**Temporary contraceptive Used (n = 209)**		
Depo-provera injection	75	35.6
Condom	51	24.5
Pills	50	24.0
Copper-T	9	4.3
Implant	9	4.3
Calendar method	12	5.8
Withdrawal	3	1.5
**Reasons for not using contraceptive (n = 26)**		
Desire for another child	13	50.0
Husband refusal	11	42.4
Adverse effects	2	7.6
**Had desire for additional child (n = 280)**		
Yes	89	31.8
No	191	68.2
**Desired sex of additional children(n = 89)**		
Son	55	61.8
Daughter	25	28.1
Either	9	10.1

### Fertility behavior of respondents’

Out of total respondents 90.7% were currently using contraceptive and 9.3% were not using any method of contraceptive. Among the users 82.3% were using temporary method of contraceptive and 17.7% were using permanent method of contraceptive. Similarly, 35.6% were using depo-provera injection and 1.5% was adopting withdrawal method and among the non-users 50.0% were not using for desire for another child. On the other, majority of husband (43.9%) made decision about number of children in the family and only 21.1% respondentsmade decision about the number of children in family. In the same way, around 40% respondents discussed with their spouse about family planning method. Regarding desire for additional children, 68.2% women had no desire for additional children and 31.8% had desire for additional children. Among those who desire for additional children 61.8% desired for son and 28.1% desired for daughter (Table 3).

**Table 3:** Fertility behavior of respondents’.

Fertility Behavior	Number	Percentage
**Current use of contraceptive (n = 280)**		
Yes	254	90.7
No	26	9.3
**Contraceptive methods Used (n = 254)**		
Temporary	209	82.3
Permanent	45	17.7
**Temporary contraceptive Used (n = 209)**		
Depo-provera injection	75	35.6
Condom	51	24.5
Pills	50	24.0
Copper-T	9	4.3
Implant	9	4.3
Calendar method	12	5.8
Withdrawal	3	1.5
**Decision maker for number of children**		
Husband	123	43.9
Wife	59	21.1
Both(husband and wife)	75	26.8
Other family member	23	8.2
**Discuss with spouse about measure of FP**		
No	110	39.3
Yes	170	60.7
**Reasons for not using contraceptive (n = 26)**		
Desire for another child	13	50.0
Husband refusal	11	42.4
Adverse effects	2	7.6
**Had desire for additional child (n = 280)**		
Yes	89	31.8
No	191	68.2
**Desired sex of additional children(n = 89)**		
Son	55	61.8
Daughter	25	28.1
Either	9	10.1

### Logistic regression analysis of respondents’

#### Gender preference and socio-demographic characteristics

Respondents who were from Bramhin/Chhetri ethnic group were 60.5% (AOR = 0.395, 95% CI: 0.169–0.927)lesslikely to have gender preference compared to other group. Likewise, respondents whose husbands were literate were 71.3% (AOR = 0.287, 95% CI: 00.130–0.634)less likely to have gender preference compared to those who had illiteratehusbands (Table 4).

**Table 4:** Logistic regression analysis of respondents’ gender preference and selected socio-demographic variables. n = 280.

Characteristics	Unadjusted OR (95% CI)	Adjusted OR (95% CI)	p-value
**Ethnicity**			
Janajati	0.969 (0.506–1.854)	0.886 (0.456–1.719)	0.719
Bramhin/Chhetri	0.386 (0.166–0.896)	0.395 (0.169–0.927)	0.033*
Others	1	1	
**Educational status of husband**			
Literate	0.264 (0.121–0.577)	0.287(0.130–0.634)	0.002*
Illiterate	1	1	

*1*- *Reference category*

* *Significance at 95% CI*

#### Logistic regression analysis of temporary method of contraceptive use and selected socio-demographic variables of the respondents

Respondents who were less than 30 years of age were 59.3% (AOR = 0.407, 95% CI: 0.205–0.808) less likely to use temporary method of contraceptive compared to those who were equal or above 30 years. It may be because of their parenting desire (Table 5).

**Table 5:** Logistic regression analysis of temporary method of contraceptive use and selected socio-demographic variables of the respondents. n = 280.

Characteristics	Unadjusted OR (95% CI)	Adjusted OR (95% CI)	p-value
**Age group (years)**			
<30	3.372(1.878–6.053)	0.407(0.205–0.808)	0.010*
≥30	1	1	
**Educational status of respondents**			
Literate	2.666(1.492–4.761)	1.463 (0.717–2.982)	0.296
Illiterate	1	1	
**Educational status of husbands**			
Literate	2.531(1.161–5.520)	1.365(0.560–3.327)	0.494
Illiterate	1	1	
**Number of living children**			
≤2	2.170(1.238–3.803)	1.315(0.700–2.473)	0.395
>2	1	1	

1- Reference category

* Significance at 95% CI

### Logistic regression analysis of permanent method of contraceptive use and selected socio-demographic variables of respondents

Respondents who were30 years of age and above were 9.387 (AOR = 9.387, 95% CI:3.012–29.259)times more likely to use permanent method of contraceptive compared to those who were less than 30 years (Table 6)

**Table 6:** Logistic regression analysis of permanent method of contraceptive use and socio-demographic characteristics of respondents. n = 280.

Characteristics	Unadjusted OR (95% CI)	Adjusted OR (95% CI)	p-value
**Age group (years)**			
<30	1	1	
≥30	13.837(4.800–39.890)	9.387 (3.012–29.259)	<0.001*
**Educational status of respondent**			
Literate	1	1	
Illiterate	5.000(2.566–9.764)	2.214 (0.996–4.923)	0.051
**Educational status of husband**			
Literate	1	1	
Illiterate	3.071(1.327–7.107)	1.032 (0.393–2.712)	0.949
**Number of living children**			
≤2	1	1	
>2	2.854(1.487–5.478)	1.142 (0.549–2.377)	0.722

1- Reference category

* Significance at 95% CI

#### Logistic regression analysis of respondent’s desire for additional children and socio-demographic characteristics

Respondents who were less than 30 years of age were 6.813 (AOR = 6.813, 95%CI: 3.250–14.281)times more likely to have desire for additional children compared to those who were30 years and above. Similarly, respondents who had less than or equal to2 2 children were 5.875 (AOR = 5.875, 95% CI: 2.148–16.068)times more likely to have desire for additional children compared to those who had more than 2 children (Table 7).

**Table 7:** Logistic regression analysis of respondent’s desire for additional children and socio-demographic characteristics. n = 280.

Characteristics	Unadjusted OR (95% CI)	Adjusted OR (95% CI)	p-value
**Age group (years)**			
<30	11.878 (6.132–23.006)	6.813 (3.250–14.281)	<0.001*
≥30	1	1	
**Educational status of respondent**			
Literate	6.043(2.641–13.828)	2.058 (0.695–6.095)	0.193
Illiterate	1	1	
**Educational status of husband**			
Literate	3.348(1.132–9.907)	0.644 (0.155–2.669)	0.544
Illiterate	1	1	
**Number of living children**			
≤2	12.371(4.800–31.884)	5.875 (2.148–16.068)	0.001*
>2	1	1	

1- Reference category

* Significance at 95% CI

## Discussion

The community-based cross sectional study was designed to identify the gender preference and fertility behavior of married women in a community in far western Nepal. The findings of this study showed that the average age of the respondents was 30.23 years, with higher percentage belonging to Janajati ethnic group. Among the respondents, 74.3% were literate and 89.3% of their husbands were literate. Likewise, 55.7% respondents had monthly income of NRs 10000–20000 and 41.1% had 2 living children. Among those who had two or more children, 67.2% had both sexes. More than 90% respondents were currently using contraceptives, and 9.3% were not using any contraceptive method. Among the users, 82.3% were using temporary contraceptive methods, and 17.7% were using permanent methods.Out of 280 respondents, son preference was higher (52.9%) followed by no preference of the specific gender (31.8%). Daughter preference was observed only in 15.4% respondents. Gender preference for a male child is a well-known phenomenon in a Nepalese society. Nepal demographic and health survey report of 2022 indicate higher birth ratio of male child than females indicating strong son preference in Nepalese society [16].

The multivariate logistic analysis from this study revealed that ethnicity was important factor associated with the gender preference. Respondents who were from Bramhin/Chhetri group were 0.395times lesslikely to have gender preference compared to those in others groups. The findings also revealed that respondents whose husbands were literate were 0.287 times less more likely to have gender preference than those whose husbands were illiterate. A similar finding was reported in multi-country study, which showed that respondents belonging to the middle, second richest, and richest quintiles relative to the poorest quintiles decreases the odds of having son preference by 4%, 9% and 14% respectively [22]. Similarly, this finding is consistent with astudy conducted in Malawi on the influence of gender preference and sex composition of surviving children on childbearing intention among high-fertility married women in stable unions [23]. However, this finding is inconsistent with the study conducted rural India [24].

The multivariate logistic analysis of this study showed that the use of temporary contraceptive methods was 0.407times less likely among respondents who were less than 30 years of age,and respondents who were 30 years and above were 9.387 times more likely to adopt permanent contraceptives. This finding is similar to a study conducted in Sonapur VDC of Eastern Nepal, whichshowed that respondents in the 15–24 years age group were 3.656 times more likely to use temporary methods and 0.143 times less likely to adopt permanent contraceptive methods [17]. Similarly, the bivariate logistic analysis from this study showed that the use of temporary method was 2.531 times more likely among respondents whose husband were literate, and the use of permanent method was 3.071 times more likely among respondents whose husbands were illiterate.This demonstrates that educated people are more committed to temporary family planning methods, and this behavior is widespread among literate individuals today. This may be because they have more faith in long-acting temporary family planning techniques.A study conducted in Nigeria showed that the education of the husband was an important determinant of current contraceptive use (coefficient-0.28596, significant at 0.05 levels) [25].

Various factors, such as sex of the children (either male or female), the total number of living children, and the sex of the last child, affect the overall use of contraceptive methods. The educational level, awareness, and motivation of the husband and wife also affect the use of birth control measures. Cost is not important factor, as free family planning services are available in Nepal. Gender preference, specifically an adequate number of sons, affects the overall use of family planning methods.Women who have more sons are more likely to use sterilization, a long acting, permanent family planning method, compared to women with more daughters [26, 27]. Son preference is an important factor among other socio-demographic determinants of fertility differentials [28].

The multivariate analysis showed that the desire for additional children was 6.813 times higher among those who were less than 30 years of age. Likewise, the desire for additional children was 5.875 times more likely among respondents who had two or fewer children. This findings is consistent to the study conducted in India [29].

The limitation of the study includes potential under or over reporting of family planning method use, as it was self-reported. It was a cross-sectional study conducted among minimum number of married women, so further analytical and interventional studies needs to be conducted to investigate gender preference and fertility behavior among married women.

## Conclusion

Gender preference for children was inclined towards son preference. Ethnicity and the educational status of husband were strong predictors of gender preference. Awareness programs on gender related issues should be tailored individually targeting specific ethnic groups. Respondents who were below thirty years of age should be encouraged touse family planning methods. Similarly, family planning programs should incorporate gender issues to increase the use of family planning methods. There is no universal opinion about gender preference. Future major replications of research should include policymaking, drug-induced, biomedical methods, and illegal sex-selective abortion.Even if the study area is technologically underdeveloped, low on the socioeconomic scale, and illiterate, it can be interesting to investigate how these traditional and modern biomedical techniques of illicit sex selective abortions come to be available to married couples.

## Supporting information

S1 Appendix Questionnaire. (DOCX)

S1 Text Plain english summary. (DOCX)
