# Supplementary material for: Gender preference and fertility behavior among married women: A community based study from far western Nepal
Source: PLOS Glob Public Health. 2024 Jun 6;4(6):e0001080. doi: 10.1371/journal.pgph.0001080 (PMC11156364; doi:10.1371/journal.pgph.0001080)
Supplement: S1 Appendix — (DOCX) [file pgph.0001080.s001.docx]

**Gender preference and fertility behavior among married women in community of far western Nepal**

Respondent no:…………… Ward no:……… Date:……………………

**Direction:** Researcher ticks (✓) for the appropriate answer provided to the close ended questions according to respondent view point and fills the answer within the space provided to the open ended questions.

**Part I: Socio-Demographic Information**

| **SN** | **Questions** | **Answers & Options/code** | **Skip to** | **Remarks** |
| --- | --- | --- | --- | --- |
| 1. | What is your age?  (completed years) | ………….. |  |  |
| 2. | What is your educational status? | 1.Literate  2. Illiterate | Q. 4 |  |
| 3. | What is your level of education? | 1. General literate  2. Basic education  3. Secondary level  4. Higher Secondary  5. Bachelors and above |  |  |
| 4. | What is your husband’s educational status? | 1.Literate  2. Illiterate | Q. 6 |  |
| 5. | What is his level of education? | 1. General literate  2. Basic education  3. Secondary level  4. Higher Secondary  5. Bachelors and above |  |  |
| 6. | What is your religion? | 1. Hindu  2. Buddhist  3. Christian  4. Muslim |  |  |
| 7. | What is your ethnicity? | 1. Dalit  2. Janajati  3. Madhesi  4. Muslim  5. Brahmin/Chhetri  6. Others |  |  |
| 8. | What is your occupation? | 1. Agriculture  2. Business  3. Service  4. Daily wage  5. Housewife  6. Other (specify)……… |  |  |
| 9. | What is your husband’s occupation? | 1. Agriculture  2. Business  3. Service  4. Daily wage  5. Housework  6. Other (specify)……… |  |  |
| 10. | What is the monthly income of your family? | Rs………………. |  |  |
| 11. | What is the total number of your children? | ………….. |  |  |

**Part-II: Questions related to gender preference and fertility behavior**

| **SN** | **Questions** | **Answers & Options/code** | **Skip to** | **Remarks** |
| --- | --- | --- | --- | --- |
| 12. | Would you like to have another child? | 0. No  1. Yes | *Q.14*  *Q. 13* |  |
| 13. | If desire to have another child, what do you want? | 1. Son  2. Daughter  3. Either |  | *Do not read options* |
| 14. | Who makes decision for determining the number of children in the family? | 1. Wife  2. Husband  3. Both  4. Other family members |  |  |
| 15. | What is your desired number of children? | ………………. |  |  |
| 16. | How many of these children would you like to be son, how many would you like to be daughter? | 1. Son………….  2. Daughter……… |  |  |
| 17. | What was your preference for the first child? | 1. Son  2. Daughter  3. Either |  | *Do not read options* |
| 18. | Which child do you prefer more? | 1. Son  2. Daughter  3. Both | *Q. 19*  *Q.20*  *Q. 21* | *Do not read options.* |
| 19. | Why do you prefer a son? | 1. Land/ property inheritance  2. For obtaining dowry  3. Support in old age  4. Practical help in day to day life  5. Social status  6. To perform funeral rites  7. Others (Specify)………………. | | *Possibility of multiple response, do not read option.* |
| 20. | Why do you prefer a daughter? | 1. Help in childcare  2. Help in household chores  3. For companionship  4. To enjoy festivals  5. Others(Specify)………………. | | *Possibility of multiple response, do not read option.* |
| 21. | Do you discuss about FP measures with your spouse? | 0. No  1. Yes | *Q. 23* |  |
| 22. | If yes, what matters do you discuss? | 1. Birth interval  2. Method of contraception  3. No. of children  4. Others (Specify)……………… | | *Possibility of multiple response* |
| 23. | Are you currently doing something or using any method to delay or avoid getting pregnant? | 0. No  1. Yes | *Q.24*  *Q. 25* |  |
| 24. | If no, what is the reason you are not using family planning method? | ……………………… |  |  |
| 25. | If Yes, Which method are you using now? | ……………………… |  |  |

Thank you for your co-operation.
