# Supplementary material for: Gender preference and fertility behavior among married women: A community based study from far western Nepal
Source: PLOS Glob Public Health. 2024 Jun 6;4(6):e0001080. doi: 10.1371/journal.pgph.0001080 (PMC11156364; doi:10.1371/journal.pgph.0001080)
Supplement: S1 Text — (DOCX) [file pgph.0001080.s002.docx]

**Plain English Summary**

Gender preference leads to low contraceptive use and parity progression, which, in turn, increases morbidity and mortality among women. This study aims at identifying gender preference and fertility behavior, including contraceptive use and the desire for additional children, among married women. A cross-sectional descriptive study was conducted, and 280 households were selected using a systematic random sampling technique. One respondent from each household was interviewed using semi-structured interview schedule. The collected data were analyzed using descriptive and inferential statistics. Of 280 respondents, 44.6% aged 26-35 years, with a mean age was 30.23±7.39 years. Regarding education level, 74.3 % were literates, and 70% were paid workers. Among the respondents, 53% preferred sons, 15.4% preferred daughters, and the major reason for son preference was support in old age (87.2%). Gender preference was 0.395 times less likely in Bramhin/Chhetri ethnic group (p= 0.033) and 0.287times less likely with literate husband (p= 0.002). Regarding fertility behavior, contraceptive use was 90.7% and 31.8% of respondents had desire for additional children. The use of permanent contraceptive methods was 9.387 times more likely above 30 years (p=<0.001) and statistically significant with the education level of respondent and husband, sex composition of children, the number of children, and having preferred child. The desire for additional children was 6.813 times more likely below 30 years of age (p=<0.001) and 5.875 times more likely among those with one or two living children (p=0.001). This desire was statistically significant with the education levels of the respondent and her husband, as well as the number of living children. The study concluded that gender preference was prevalent in study area among illiterate individuals, with sons being preferred over daughters. Contraceptive use was low among respondents below 30 years of age. Enhancing educational status can reduce gender preference, and focusing family planning programs to women below 30 years of age can improve the utilization of family planning services in this age group.
